# Abnormal expression profile of plasma-derived exosomal microRNAs in patients with treatment-resistant depression

**DOI:** 10.1186/s40246-021-00354-z

**Published:** 2021-08-21

**Authors:** Lian-Di Li, Muhammad Naveed, Zi-Wei Du, Huachen Ding, Kai Gu, Lu-Lu Wei, Ya-Ping Zhou, Fan Meng, Chun Wang, Feng Han, Qi-Gang Zhou, Jing Zhang

**Affiliations:** 1 Department of Clinical Pharmacology, School of Pharmacy, Nanjing Medical University, Nanjing, 211166 Jiangsu Province China; 2 Nanjing Brain Hospital Affiliated to Nanjing Medical University, Nanjing, 210029 Jiangsu Province China; 3 Functional Brain Imaging Institute of Nanjing Medical University, Nanjing, 210029 Jiangsu Province China; 4 Sir Run Run Hospital, Nanjing Medical University, Nanjing, 211167 Jiangsu Province China; 5 Key Laboratory of Cardiovascular and Cerebrovascular Medicine, School of Pharmacy, Nanjing Medical University, Nanjing, 211166 Jiangsu Province China

**Keywords:** Depression, Exosomes, MicroRNAs, Biomarker, High-throughput sequencing

## Abstract

Whether microRNAs (miRNAs) from plasma exosomes might be dysregulated in patients with depression, especially treatment-resistant depression (TRD), remains unclear, based on study of which novel biomarkers and therapeutic targets could be discovered. To this end, a small sample study was performed by isolation of plasma exosomes from patients with TRD diagnosed by Hamilton scale. In this study, 4 peripheral plasma samples from patients with TRD and 4 healthy controls were collected for extraction of plasma exosomes. Exosomal miRNAs were analyzed by miRNA sequencing, followed by image collection, expression difference analysis, target gene GO enrichment analysis, and KEGG pathway enrichment analysis. Compared with the healthy controls, 2 miRNAs in the plasma exosomes of patients with TRD showed significant differences in expression, among which has-miR-335-5p were significantly upregulated and has-miR-1292-3p were significantly downregulated. Go and KEGG analysis showed that dysregulated miRNAs affect postsynaptic density and axonogenesis as well as the signaling pathway of axon formation and cell growths. The identification of these miRNAs and their target genes may provide novel biomarkers for improving diagnosis accuracy and treatment effectiveness of TRD.

**Supplementary Information:**

The online version contains supplementary material available at 10.1186/s40246-021-00354-z.

## Introduction

Depression has been a common mental illness and major public health problem. Its core symptoms are characterized by low mood and anhedonia. Depression seriously reduces the efficiency of patients’ work and quality of life and even leads to suicide. In 2008, the World Health Organization (WHO) listed major depression as the third leading cause of the global burden of disease, and it is predicted that the condition will rank first by 2030 [1]. Although many researchers focus on this field, the understanding of the pathophysiology of depression is still limited. At present, the pathological mechanisms of depression mainly include the abnormal metabolism of serotonin (5-HT), norepinephrine (NE), and other monoamine neurotransmitters, hypothalamic-pituitary-adrenal (HPA) axis hyperfunction, neuroinflammation, and impaired synaptic plasticity, etc. [2–5]. At present, selective serotonin reuptake inhibitors (SSRIs) are still widely used as first-line treatments for depression; however, 30% or more of patients fail to get response to pharmacologic interventions and have repeated depressive episodes, diagnosed as treatment-resistant depression (TRD) [6]. In recent years, the research on biomarkers of depression has become a new hotspot. Biomarker research would not only hold promise in individualized diagnosis and treatment, but also enhance treatment responsivity in TRD.

Exosomes are extracellular vesicles with a lipid bilayer and a diameter of 40–200nm. They are widely distributed in the blood, cerebrospinal fluid (CSF), urine, and other body fluids. Many cells in an organism, including neurons, can secrete exosomes [7]. The bilayer membrane structure of exosomes allows them to bind to the cell membrane and readily cross the blood-brain barrier (BBB) to regulate the function of the nervous system [8]. There are various biologically active molecules, including lipids, proteins, and small RNA in exosomes, which could be transported to nearby or distant cells. Exosomes released by the invagination of intracellular lysosomal micro-particle in the central nervous system (CNS) may play an active role in the occurrence or development of diseases in the CNS such as Alzheimer’s disease (AD), Parkinson’s disease (PD), prion disease, multiple sclerosis (MS), schizophrenia (SCZ), and brain tumors [9]. The exosome translocation between cells participates in the communication between cells and regulates the function of target cells. A large number of studies have shown that exosomes play an important role in the communication of the CNS, neural regeneration, synaptic plasticity, and neuroinflammation [10–12].

MicroRNA is a small non-coding RNA, about 22 nucleotides in length, which can directly interact with the seed complementary sequence in the 3′ untranslated regions (3′-UTR) of the target messenger RNA (mRNA), thus inhibiting the expression of the target gene. Compared with other biological fluids such as plasma and saliva, many miRNAs exist in exosomes [13]. The lipid bilayer structure of exosomes could protect miRNAs, making them more stable than free miRNAs. Many studies have shown that miRNAs is widely involved in regulating various systems, including the nervous system, and may play a vital role in brain function, especially in neurogenesis, neuronal development, and synaptic plasticity [14]. Studies have shown that miRNAs expression in exosomes can be changed in different disease states, making the miRNAs the candidates as the biomarker for the pathogenesis of CNS diseases [15].

In view of the regulatory role of exosomes in neuroinflammation, neurogenesis, and plasticity, exosomes have long been hypothesized to be involved in the psychopathological processes of mental disorders. Banigan et al. detected miRNAs changes in exosomes in frozen postmortem prefrontal cortices of patients with SCZ and bipolar disorder and found that microRNA-497 (miR-497) increased in SCZ patients and miR-29c upregulated in bipolar patients [16]. Du et al. conducted the genome-wide miRNA expression profile analysis on serum-derived exosomes from 49 first-onset, drug-free SCZ patients, and 46 normal persons and found 11 miRNAs showed a significant difference between the two groups, which can be used to classify samples from SCZ patients and control subjects with nearly 90% accuracy in the training samples, and approximately 75% accuracy in the testing samples [17]. Recently, some researches have indicated that the exosomes and associated miRNAs may contribute to the inflammatory mechanisms underlying depression [18]. The exosome-derived miR-139-5p had the potential to be a biomarker for major depressive disorder [19]. Furthermore, studies have shown that exosomes from patients with major depression cause depression-like behaviors in mice involved in miR-139-5p-regulated neurogenesis [20]. Bone marrow mesenchymal stem cell-derived exosomes upregulated miR-26a to boost hippocampal neuron proliferation and suppressed apoptosis in depressed rats [21], and NK cell-derived exosomes could carry miR-207and alleviate depression-like symptoms in mice [22]. These results indicate that exosomes are involved in the occurrence and development of depression and other mental disorders. However, the potential role of exosomes in the pathophysiology of treatment-resistant depression (TRD) is still poorly understood. Given that exosomes are involved in psychopathological processes, including neuroinflammation, neurogenesis, plasticity, and epigenetic regulation [23], exosomes are likely to play an important role in the pathogenesis of depression.

This study collected the peripheral blood samples from 4 TRD patients and 4 healthy controls and extracted miRNAs for high-throughput sequencing also known as next-generation sequencing (NGS). We try to find the different major miRNAs in the plasma exosomes of TRD patients and their function in different signal pathways. The recognition of these miRNAs and their target genes may provide a new high-throughput screening platform for the early detection of the severity of depression and the treatment response, which will improve the diagnosis accuracy and treatment effectiveness of the depression.

## Materials and methods

### Study participants

The inclusion criteria for the patient with TRD were as follows [24]: (1) 18–65 years old; (2) recent diagnosis of depression by ICD-10 criteria for a mood disorder and depressive episode; (3) diagnosis of antidepressant resistance: recurrent attacks in recent 3 years after two or more depressants treatment; (4) no history of taking any other drugs; and (5) the total score of the Hamilton Depression Scale (HAM-D) was higher than 17, indicating mild to moderate depression. Before treatment, the patients were re-evaluated with HAM-D. At the same time, blood samples were collected simultaneously with symptom self-evaluation and before treatment. Subjects in the healthy control group matched the age of patients with depression; they (1) had no history of depression, (2) had a HAM-D score of 0-4 for more than 8 weeks, and (3) exclude any of the conditions described in the criteria. Participants (healthy controls and depressed patients) were excluded from the study if they had severe personality disorders, risk of suicide, pregnancy, thyroid disease, use of any psychotherapy, and participation in any other psychiatric intervention study.

This study was approved by the Nanjing First Hospital Organization evaluation committee, showed in supplementary information. All eight participants provided informed consent before signing up.

### Sample collection

Plasma samples of depression patients and healthy control participants were collected before treatment. At least 4 ml of venous blood was collected from the subjects’ tubes with EDTA anticoagulant. After 10 min of centrifugation at 3000 rpm at room temperature, plasma samples (ideally 2 ml/sample) are stored in RNase antifreeze tubes at −80°C.

### Exosome isolation and identification

Exosomes were isolated using ExoEasy Maxi Kit (Qiagen, 76064, USA). Briefly, 2 ml plasma samples filtered using a 0.8 μm filter syringe (Millipore, SLAA033SB, USA) are mixed with Buffer XBP and bound to an exoEasy membrane affinity spin column. The bound exosomes are washed with buffer XWP, eluted with 400μl Buffer XE (an aqueous buffer containing primarily inorganic salts). The exosome samples were stored at −20°C until further detection. Exosomes were observed by transmission electron microscopy (TEM) (FEI, Tecnai Spirit TEM T12). The size and concentration of the exosomes were assessed using Flow NanoAnalyzer (NanoFCM, Xiamen, China). Exosomal proteins CD81 (BD, 551108, USA) and CD9 (BD, 555317, USA) expression were measured using Nano-Flow analysis (NanoFCM, Xiamen, China).

### Exosome RNA extraction

According to the manufacturer’s instructions, using exoRNeasy Midi Kit (Qiagen, 77144, USA), separate the exosome RNA, including miRNA. In brief, exosome samples are homogenized in QIAzol Lysis Reagent, chloroform is added to the QIAzol eluate, and the aqueous phase is recovered and mixed with ethanol. Total RNA, including miRNA, binds to the spin column, washed three times, and eluted. Use Agilent 2100 Bio-analyzer (Santa Clara, CA, USA) to analyze the yield and quality of the separated RNA (Supplement figure 1).

### Next-generation sequencing analysis

RNA fragments containing 17–30 nucleotides, or 15–35 nucleotides, were isolated by gel purification. A random primer is attached to both ends of the isolated RNA fragment and then reverse transcribed into the cDNA as previously described [25]. Amplification was carried out by PCR to establish a sequence library, and Illumina HiSeq high-throughput sequencing was carried out using the SE50 strategy [25]. The expression level of miRNA was obtained by Qiagen online platform. Edger package [26] was compared to those in the genome to get information about the location of known mature and precursor miRNAs in the genome. Clean reads in the genome were matched to the location of miRNA [27] and further analyzed its expression, sequence, and structure. As mentioned above, the target genes that differentially expressed miRNAs are predicted and sought by miRanda (http://www.microrna.org/microrna/home.do), RNAhybrid (http://bibiserv.techfak.uni-bielefeld.de/rnahybrid/), and miRDB (http://www.mirdb.org/) database. The target genes of differentially expressed miRNAs were subjected to gene ontology (GO) and Kyoto encyclopedia of genes and genomes (KEGG) analyses as previously described [28, 29]. Gender-specific and sex hormone-regulated miRNAs were excluded from the study.

### Statistical analysis

Unpaired Student’s *t* test with Welch’s correction was used to estimate the differences between groups. All statistical analysis is performed by using primer 8 software (Cary, NC, USA). When the *p*-value is less than 0.05, these results are considered significant.

## Results

### Identification of extracted plasma exosomes

**Fig. 1:**
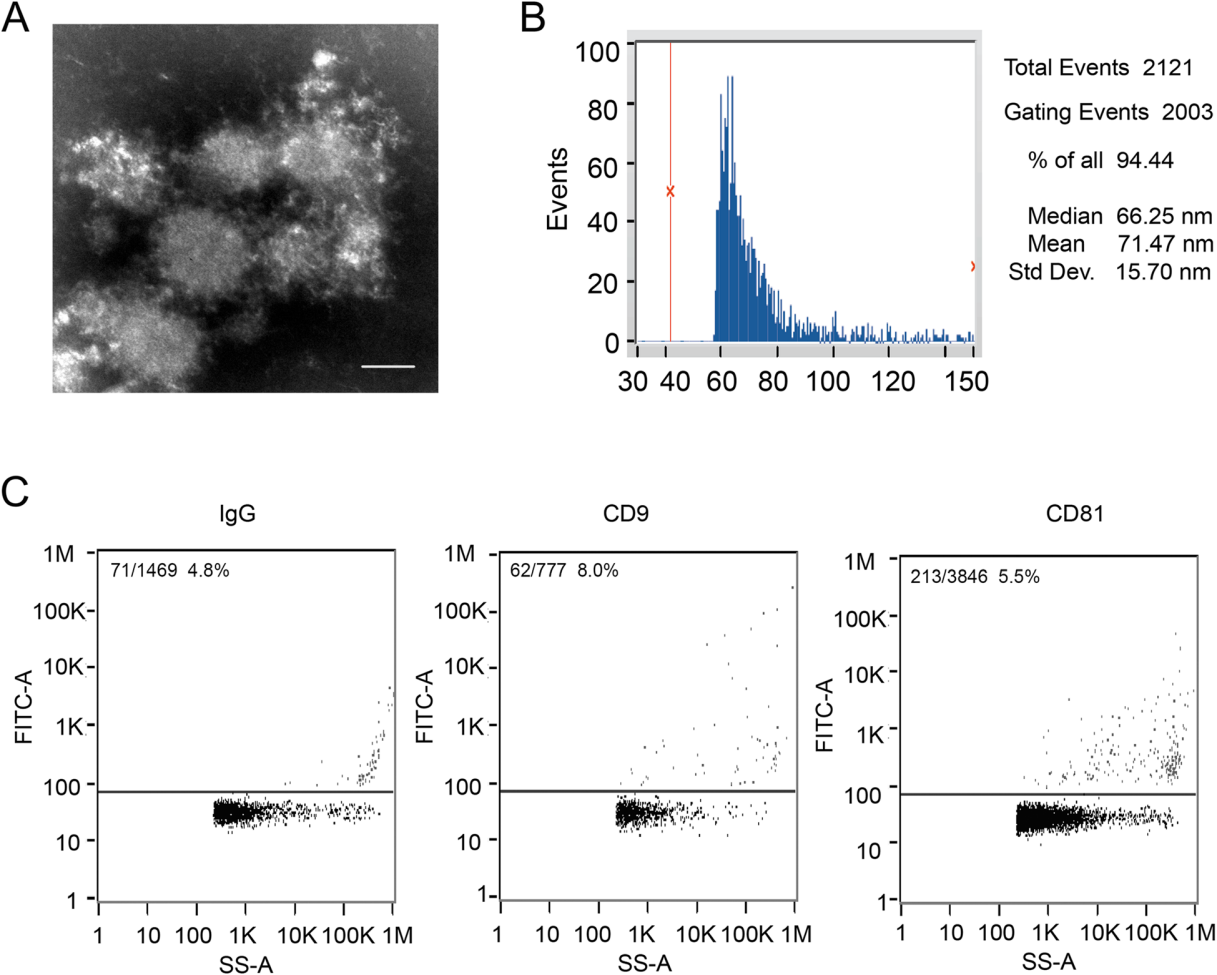
The morphology and characteristics of extracted exosomes from patients with TRD and health controls. **A** The transmission electron micrograph of plasma-derived exosomes. Scale bar: 100nm. **B** The particle size distribution of plasma-derived exosomes. **C** The fluorescence of exosomal proteins (CD9 and CD81) was detected by flow cytometry

As shown in Fig. 1, the exosomes are round or oval vesicles with different sizes (30–100nm) under the electron microscope, which are composed of the outer membrane with deeper staining and the inner layer with shallow staining and lower electron density by the description of exosomes reported in the literature [30]. The size of exosomes ranged from 71 to 79 nm, and the expression of CD9 and CD81 was positive.

### Identification of abnormally expressed miRNAs in patients with depression by NGS

**Fig. 2:**
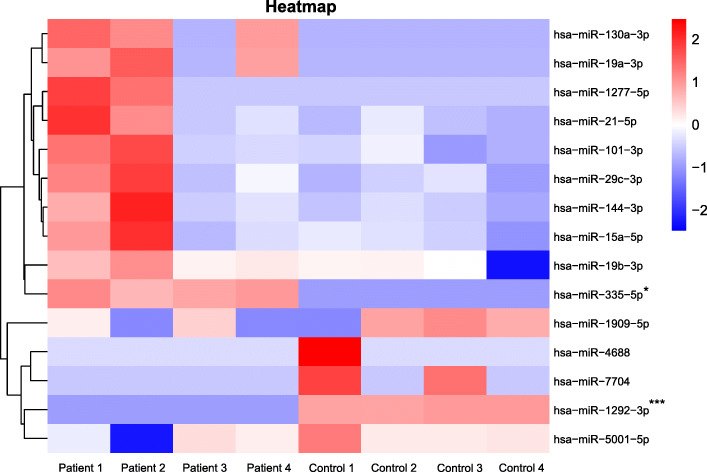
Heatmap of differentially expressed miRNAs. This figure shows the distribution of 15 different miRNAs in four depression patients and four healthy controls. Values below 0 are superimposed in blue, and the smaller the number, the darker the color. Values above 0 in red and the larger the value, the darker the color. *P<0.05, ***P<0.001, unpaired Student’s *t*-test with Welch’s correction

The study included four patients with major depressive depression and four healthy controls. Using high NGS, the analyzed miRNAs are considered candidate miRNAs if they are upregulated or downregulated ≥ 2 times, and the adjusted *p*-value was < 0.05. As shown in the heatmap of differential miRNAs expression in Fig. 2, there were 10 upregulated miRNAs: has-miR-19a-3p, has-miR-144-3p, has-miR-130a-3p, has-miR-335-5p, has-miR-101-3p, has-miR-1277-5p, has-miR-15a-5p, has-miR-29c-3p, has-miR-19b-3p, and has-miR-21-5p and 5 down-regulated miRNAs: has-miR-7704, has-miR-1292-3p, has-miR-1909-5p, has-5001-5p, and has-miR-4688 were identified by NGS analysis (Supplementary Table 1). We further used unpaired Student’s *t* test with Welch’s correction to analyze the expression levels of these 15 microRNAs and found that has-miR-335 and has-miR-1292 still had statistical differences (Table 1). Therefore, we selected has-miR-335 and has-miR-1292 as candidate microRNAs for subsequent analysis.

**Table 1:** Significantly dysregulated miRNAs in plasma exosomes in TRD patients compared with healthy controls

MicroRNA	Fold-change	Up/down	Adjusted ***p***-values
Has-miR-335-5p	149.231458	Up	0.0315
Has-miR-1292-3p	0.007057	Down	0.0006

Adjusted *p*-values were determined using unpaired Student’s *t*-test with Welch’s correction

### Analysis of differentially expressed miRNAs targeted genes

Based on the 2 miRNAs with significant expression differences, all target genes in miRanda, RNAhybrid, and miRDB database were predicted [31, 32].

### GO analysis of miRNAs differentially expressed in exosomes of patients with depression

Gene ontology (http://www.geneontology.org/) functional enrichment analysis of miRNAs target genes can show the functional enrichment of miRNAs target genes and clarify the differences among samples at the gene function level. As shown in Table 2, GO analyses the differentially expressed miRNAs are related to cell composition, biological process, and molecular functions. Cell composition includes the regulation of postsynaptic density and transcription regulators. Biological process includes the axonogenesis. The regulation of molecular functions consists of the post-transcriptional change.

**Table 2:** GO analysis of the candidate miRNAs in TRD

Term	Overlap	***P***-values
Axon part (GO:0033267)	32/415	0.003757
CHD-type complex (GO:0090545)	4/18	0.008459
Cytoplasmic ribonucleoprotein granule (GO:0036464)	23/268	0.003642
Early endosome (GO:0005769)	34/434	0.002231
NuRD complex (GO:0016581)	4/18	0.008459
PML body (GO:0016605)	13/113	0.002301
Postsynaptic density (GO:0014069)	25/35	0.006811
Ribonucleoprotein granule (GO:0035770)	23/281	0.006435
Transcription factor complex (GO:0005667)	33/403	0.001257
Transcriptional repressor complex (GO:0017053)	11/90	0.003031
Appendage development (GO:0048736)	23/188	0.000026
Axonogenesis (GO:0007409)	46/498	0.000010
Cell fate commitment (GO:0045165)	34/304	0.000003
Embryonic organ development (GO:0048568)	43/480	0.000041
Limb development (GO:0060173)	23/188	0.000026
Lung development (GO:0030324)	23/193	0.000040
Positive regulation of neuron differentiation (GO:0045666)	41/402	0.000003
Positive regulation of neuron projection development (GO:0010976)	32/304	0.000018
Regulation of cellular component size (GO:0032535)	39/407	0.000021
Regulation of transcription involved in cell fate commitment (GO:0060850)	7/18	0.000010
DNA-binding transcription activator activity, RNA polymerase II-specific (GO:0001228)	46/479	0.000009
GTP binding (GO:0005525)	33/406	0.002841
Guanyl nucleotide binding (GO:0019001)	35/429	0.002028
Guanyl ribonucleotide binding (GO:0032561)	35/429	0.002028
Nucleoside binding (GO:0001882)	33/422	0.005119
Purine nucleoside binding (GO:0001883)	33/414	0.003839
Purine ribonucleoside binding (GO:0032550)	33/411	0.003434
Ribonucleoside binding (GO:0032549)	33/415	0.003982
SUMO transferase activity (GO:0019789)	5/20	0.002265
Transcription corepressor activity (GO:0003714)	22/195	0.000214

The overlap value indicates the number of predicted downstream target genes of the candidate miRNAs identified versus the total number of genes related to the term in the GO gene expression network

### Enrichment KEGG analysis of differentially expressed miRNA target gene

KEGG database is a knowledge base for systematic analysis of gene function, an association of genome information and functional information. As shown in Table 3, KEGG analysis of the differentially upregulated and downregulated expressed miRNAs revealed some are related to MAPK, Ras, and PI3K-AKT signaling pathway.

**Table 3:** KEGG analysis of the candidate miRNAs in TRD

ID Description	Overlap	***P***-values
Steroid biosynthesis (KEGG:hsa00100)	13/20	2.156E−07
Parathyroid hormone synthesis, secretion, and action (KEGG:hsa04928)	35/106	4.265E−07
PI3K-Akt signaling pathway (KEGG:hsa04151)	77/354	3.086E−05
Ras signaling pathway (KEGG:hsa04014)	55/232	3.744E−05
Breast cancer (KEGG:hsa05224)	38/147	8.661E−05
MAPK signaling pathway (KEGG:hsa04010)	64/294	0.0001402
Calcium signaling pathway (KEGG:hsa04020)	54/240	0.0001912
Complement and coagulation cascades (KEGG:hsa04610)	24/85	0.000424
Chemokine signaling pathway (KEGG:hsa04062)	44/192	0.000483
Cytokine-cytokine receptor interaction (KEGG:hsa04060)	62/295	0.0004842

The overlap value indicates the number of predicted downstream target genes of the candidate miRNAs identified versus the total number of genes related to the term in the KEGG gene expression network

### Target gene regulated downstream signaling pathway of miRNAs differentially expressed miRNAs

**Fig. 3:**
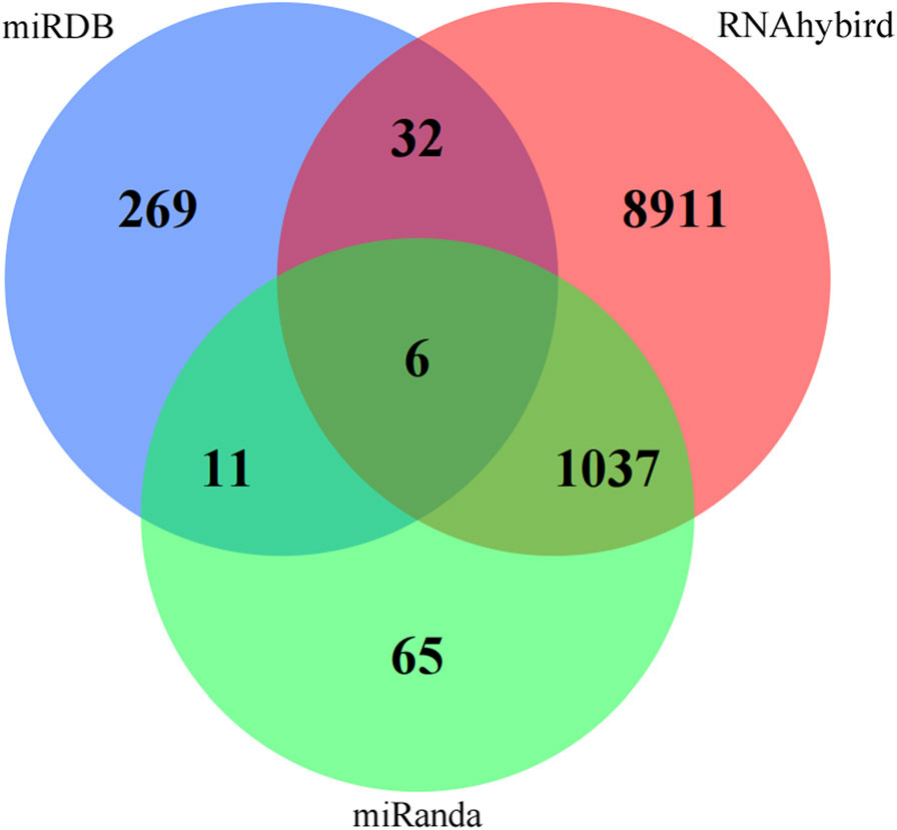
Venn diagram of target gene prediction results. This figure shows the gene prediction results by three different methods (miRanda, RNAhybrid, and miRDB). The result of miRDB is superimposed in blue. The result of RNAhybrid is superimposed in red. The result of miRanda is superimposed in green. The overlapping shows the same result in 2 or 3 different methods

miRanda, RNAhybrid, and miRDB were used to predict different miRNAs target genes, and 1086 target genes were selected as the highest related target genes (Fig. 3). We used overlapping parts from two or more databases as predictive genes and then further searched the literature to select target genes related to depression. As shown in Table 4, 14 genes were selected as target genes.

**Table 4:** Putative target genes of the differentially expressed miRNAs in plasma-derived exosomes in four patients with TRD compared to four healthy control subjects

Genes	NO. of miRNAs targeted	miRNA expression	Reference(s)	Function(s)
ADRA1B	1 (has-miR-1292-3p)	Down	[33]	GABA, serotonin
ADRB1	1 (has-miR-1292-3p)	Down	[34]	Synapsin
CACNA1C	1 (has-miR-1292-3p)	Down	[35]	Synapsin Dopamine Glutamate Adrenergic
CREB1	1 (has-miR-1292-3p)	Down	[36]	Synapsin Dopamine NMDA Glutamate serotonin
FGFR1	1 (has-miR-1292-3p)	Down	[37]	Dopamine
GNAS	1 (has-miR-1292-3p)	Down	[38]	Synapsin Glutamate serotonin
GRIK5	1 (has-miR-1292-3p)	Down	[33]	Synapsin Dopamine NMDA Glutamate GABA
HTR1A	1 (has-miR-1292-3p)	Down	[39]	Synapsin Glutamate
NOS1	1 (has-miR-1292-3p)	Down	[40]	Serotonin Dopamine NMDA Glutamate GABA Adrenergic
OPRM1	1 (has-miR-1292-3p)	Down	[41]	Synapsin Dopamine Glutamate GABA Adrenergic
P2RX7	1 (has-miR-1292-3p)	Down	[42]	Glutamate GABA
PDE10A	1 (has-miR-1292-3p)	Down	[43]	Synapsin
NUFIP2	1 (has-miR-335-5p)	Up	[44]	MDD
TCF4	1 (has-miR-335-5p)	Up	[44]	MDD

These target genes involve the synaptic synthesis and transport of many neurotransmitters, including dopamine, glutamate, norepinephrine, serotonin, and the binding process with corresponding receptors.

## Discussion

Exosomes are tiny vesicles secreted by cells. When exosomes were first found, they were considered as a way to excrete waste [45]. With the development of research, it was found that the mRNA and miRNA carried by exosomes can be translated into proteins in the cytoplasm [46]. In addition, the miRNAs transferred by exosomes also have biological activation and can be targeted and regulated by the level of mRNA in cells after entering the target cells. As a cell-cell communication medium, exosomes act on target cells in three ways: the ligands on the surface of exosomes directly bind to the receptor of target cells; the soluble components of proteins on the membrane hydrolyzed by protease bind to the receptor of target cells, and they act through the fusion or endocytosis of target cells. The function of recognizing target cells by exosomes is to avoid being randomly transmitted to other tissue cells to play a targeted therapeutic role. It can provide drug carriers for precision medicine and horizontally transfer the contents to target cells through membrane fusion to play a biological function [47, 48].

The exosomes can fuse directly with the target cell membrane and release the proteins, mRNA, and miRNAs. MicroRNA is a non-coding RNA that mediates post-transcriptional silencing of mRNA and can regulate various cellular and molecular signaling pathways. The miRNA is widely found in human blood, urine, CSF, and other body fluid samples and provides abundant, stable, sensitive, and specific biological information, which can transfer information in the circulatory system. In recent years, many studies have shown that miRNA is one of the important molecules in the pathogenesis of depression. It has been demonstrated that the deregulation of miR-16, miR-451, miR-223, miR-182, and other miRNAs may be related to the depression state of different patients [49]. These findings suggest that tissue-specific circulating miRNAs can be used as biomarkers to diagnose and treat depression. At present, the research methods of miRNA are mainly through real-time quantitative PCR and gene chip technology. These methods mainly focus on the expression and quantification of miRNA and are limited to the research of miRNA with sequence information or secondary stem ring structure information, so it is impossible to find new miRNA molecules. Based on high-throughput sequencing technology, which enables researchers to sequence miRNAs in samples with high-throughput directly, higher sensitivity, large sequencing throughput, and be able to detect rare transcripts with extremely low abundance, and it can annotate known miRNAs with a public miRNA database, and further analyze unmatched new miRNA species and isomers, and sought further research information [50, 51].

In this study, we used high-throughput sequencing technology to screen sensitive miRNAs related to depression. The results showed that the exoRNeasy serum/plasma MIDI kit was successfully used to extract plasma exosomes. Fifteen miRNAs with significant expression differences were screened by high-throughput sequencing, among which miR-19a-3p, miR-144-3p, miR-130a-3p, miR-355-3p, miR-101-3p, miR-1277-5p, miR-15a-5p, miR-29c-3p, and miR-21-5p were upregulated, miR-7704, miR-1292-3p, miR-1909-5p, miR-5001-5p and miR-4688 were downregulated. Some of these abnormal miRNAs have been reported. For example, miR-144-3p is downregulated in the plasma of patients with depression, and the expression level of miR-144-3p in the plasma is related to depression symptoms [52]. Some studies have shown that miR-144-3p can target the mRNA of glutamate decarboxylase-67 (GAD67), vesicular γ-aminobutyric acid (GABA) transporter, and GABA transporter-3; inhibit their translation; and thus destroy the release and uptake of GABA. This may constitute the subcellular and molecular mechanism of GABA reduction in patients with depression [53]. The miR-335 participates in treating severe depression by targeting glutamate metabotropic receptor 4 (GRM4) [54]. miR-15b is upregulated in the medial prefrontal cortex of depression like mice [55]. miR-101 is decreased in the ventrolateral orbital cortex (VLOs) of chronic unpredictable mild stress (CUMS) rat brain and targets dual-specific phosphatase 1 (DUSP1) to modulate depressive-like behaviors [56]. miR-101 and miR-130a were significantly downregulated in the prefrontal cortex of depressed suicide subjects [57]. These results all prove that microRNAs play a key role in the pathogenesis of depression by targeting multiple cellular and molecular pathways. We further used unpaired Student’s *t* test with Welch’s correction to analyze the expression levels of these 15 microRNAs and found that has-miR-335 and has-miR-1292 still had statistical differences. These two microRNAs have been proved to be associated with synaptic function and TRD.

Notably, compared with plasma, urine, or other biological fluids, exosomes are rich in microRNA (miRNA). Most miRNAs that can be obtained from serum and saliva are contained in exosomes, and some miRNAs seem to depend on exosomes because they are not directly detected as free-floating molecules in biological fluids [13]. However, there are still few studies on exosomal microRNAs in depression. Wei et al. explored serum exosome miRNA expression profile in patients with MDD and healthy control subjects to identify potential MDD markers by microRNA sequencing. Compared with control subjects, 24 microRNAs were upregulated and 14 were downregulated. The upregulation of miR-19a-3p was consistent with our test results [20].

GO analysis showed that the target genes of different miRNAs were mainly enriched in the postsynaptic density and axonogenesis. The changes and damages of neural plasticity were closely related to the development of depression. The decrease of synaptic formation, which is closely related to the impairment of synaptic connections between neurons [58], is related to the pathogenesis of adult depression [59]. In the KEGG pathway analysis, the PI3K-Akt signaling pathway, MAPK signaling pathway, and Ras signaling pathway are closely related to depression. MAPK signaling pathway and Ras signaling pathway are some of the downstream pathways of BDNF [60]. Brain-derived neurotrophic factor (BDNF) and its receptor are widely expressed in the CNS, which affect many functions of neurons, such as the growth, morphology, and plasticity of synaptic structure [61]. The BDNF related signaling pathway is one of the pathogenesis of depression. They exist in neurons and other brain cells and participate in growth factors, cytokines, oxidative stress, hormones, and other physical stimuli [62]. A PI3K-Akt signaling pathway is downstream of the BDNF-TrkB signaling pathway. Some studies have shown that the PI3K-Akt signaling pathway is abnormal in depression, and it plays an important role in antidepressant and synaptic protein synthesis [63, 64]. In addition, target genes selected based on differential expression of microRNAs are involved in the synaptic synthesis and transport of many neurotransmitters, including dopamine, glutamate, norepinephrine, serotonin, and the binding process with corresponding receptors [29]. A genome-wide association meta-analysis based on 135,458 cases and 344,901 controls and identified 44 independent and significant loci, including NUFIP2 and TCF4. MicroRNAs in exosomes may regulate the release and function of neurotransmitters closely related to depression by regulating synaptic terminals, affecting the function of different brain regions, and regulating depression behavior. All the above pieces of evidence indicated the potential role of exosomal miRNA in depression.

## Conclusion

In conclusion, exosomal miRNAs can regulate postsynaptic density and axonogenesis in TRD. Further studies will evaluate whether the dysregulation of any specific miRNA or combination of miRNAs has a role in the pathology of depression or whether they may represent potential markers of the disease or treatment response. In addition to the traditional direct regulation of gene transcription level, miRNAs in exosomes are closely related to the function of neuronal synapses and neurotransmitters and may be involved in regulating neurotransmitter release between different brain regions.

## Supplementary Information

**Additional file 1:** Supplement Table 1.

**Additional file 2:** Supplement Figure 1.

## Data Availability

Data should be made available from corresponding authors on reasonable request.
