# Supplementary material for: Abnormal expression profile of plasma-derived exosomal microRNAs in patients with treatment-resistant depression
Source: Hum Genomics. 2021 Aug 21;15:55. doi: 10.1186/s40246-021-00354-z (PMC8379796; doi:10.1186/s40246-021-00354-z)
Supplement: Supplementary file 1 — Additional file 1: Supplement Table 1. [file 40246_2021_354_MOESM1_ESM.docx]

**Supplementary Table 1 The raw data for NGS analysis**

| **miRNA** | **FC(Control/Patient)** | **p-value** | **Patient1-UMIs-RPM** | **Patient2-UMIs-RPM** | **Patient3-UMIs-RPM** | **Patient4-UMIs-RPM** | **Control1-UMIs-RPM** | **Control2-UMIs-RPM** | **Control3-UMIs-RPM** | **Control4-UMIs-RPM** |
| --- | --- | --- | --- | --- | --- | --- | --- | --- | --- | --- |
| hsa-miR-19a-3p | 0.002942296 | 0.001442757 | 320.8628071 | 1749.323191 | 196.6691526 | 0 | 0 | 0 | 0 | 0 |
| hsa-miR-144-3p | 0.136375566 | 0.005429769 | 3178.069708 | 15075.31923 | 911.8297075 | 711.4361045 | 650.9018142 | 869.2545407 | 707.7465241 | 474.8094458 |
| hsa-miR-130a-3p | 0.004754343 | 0.0107434 | 909.1112867 | 311.3978455 | 178.7901387 | 0 | 0 | 0 | 0 | 0 |
| hsa-miR-335-5p | 0.006700623 | 0.012878652 | 420.1774855 | 119.0638821 | 268.1852081 | 185.5920273 | 0 | 0 | 0 | 0 |
| hsa-miR-7704 | 271.7645821 | 0.013429609 | 0 | 0 | 0 | 0 | 1452.011739 | 0 | 361.0951654 | 0 |
| hsa-miR-1292-3p | 141.7131053 | 0.01375008 | 0 | 0 | 0 | 0 | 216.9672714 | 202.8260595 | 259.9885191 | 263.7830255 |
| hsa-miR-101-3p | 0.232354804 | 0.016309659 | 14148.52187 | 21477.29258 | 2735.489122 | 2536.424373 | 2620.297047 | 3433.555436 | 1545.487308 | 1899.237783 |
| hsa-miR-1909-5p | 17.71274355 | 0.01943915 | 175.7105848 | 0 | 0 | 572.2420841 | 0 | 3230.729376 | 7900.762218 | 2189.399111 |
| hsa-miR-1277-5p | 0.004263376 | 0.022608095 | 1268.172047 | 293.0803252 | 0 | 0 | 0 | 0 | 0 | 0 |
| hsa-miR-15a-5p | 0.226645429 | 0.027963844 | 2352.993919 | 6530.195995 | 625.7654855 | 448.5140659 | 717.6609746 | 651.9409055 | 563.308458 | 316.5396306 |
| hsa-miR-29c-3p | 0.267721154 | 0.02828584 | 3926.749591 | 6988.134003 | 1323.047027 | 850.630125 | 767.730345 | 956.1799948 | 1141.060723 | 633.0792611 |
| hsa-miR-19b-3p | 0.174611489 | 0.032232683 | 1191.776141 | 3489.487621 | 429.0963329 | 355.7180523 | 333.7958022 | 347.7018163 | 259.9885191 | 0 |
| hsa-miR-5001-5p | 6.557911329 | 0.036605844 | 175.7105848 | 0 | 429.0963329 | 649.5720954 | 6725.985413 | 478.0899974 | 476.6456183 | 527.5660509 |
| hsa-miR-21-5p | 0.307705154 | 0.037067539 | 37739.57779 | 19352.46022 | 6436.444994 | 5397.634793 | 4756.590181 | 6939.54875 | 5026.444702 | 4484.311433 |
| hsa-miR-4688 | 247.6390431 | 0.048146855 | 0 | 0 | 0 | 0 | 1652.289221 | 0 | 0 | 0 |
