# Supplementary material for: Abnormal expression profile of plasma-derived exosomal microRNAs in patients with treatment-resistant depression
Source: Hum Genomics. 2021 Aug 21;15:55. doi: 10.1186/s40246-021-00354-z (PMC8379796; doi:10.1186/s40246-021-00354-z)

The Report of Exosome RNA detection

The Process and Method of sample detection

1. The detection of RNA concentration: Qubit 3.0
2. The detection of RNA integrity (fragment distribution):Agilent 2.0

The result of sample detection


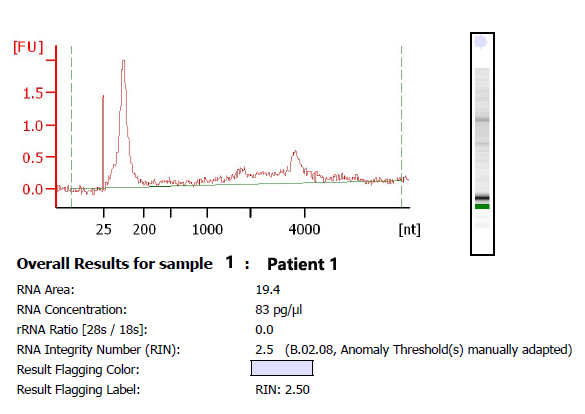


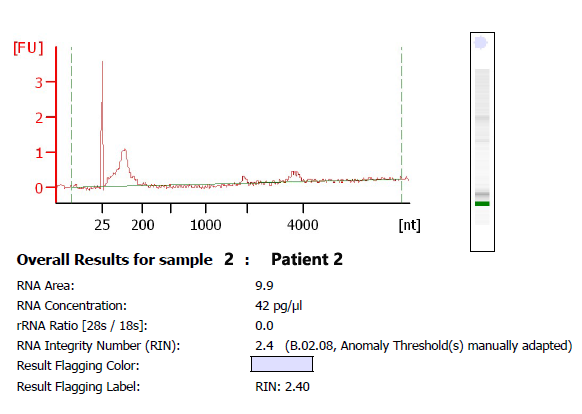


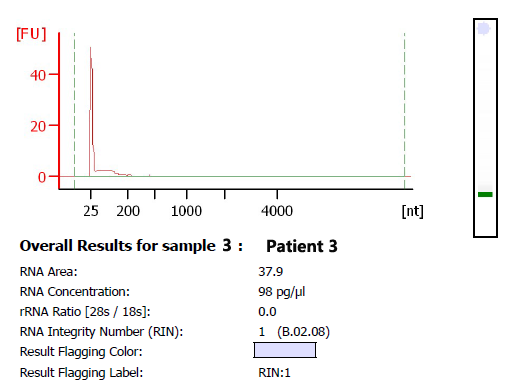


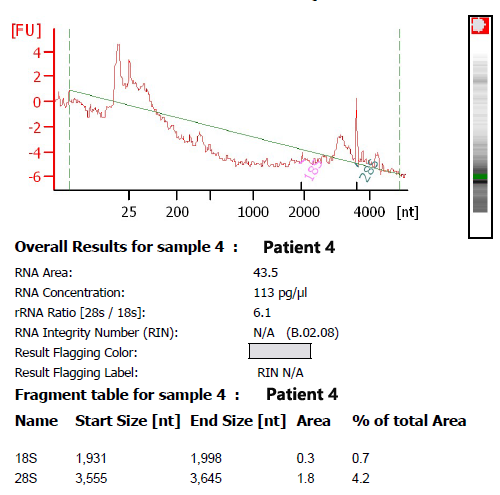


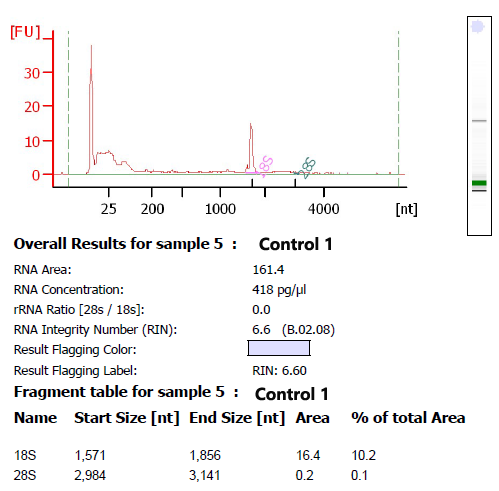


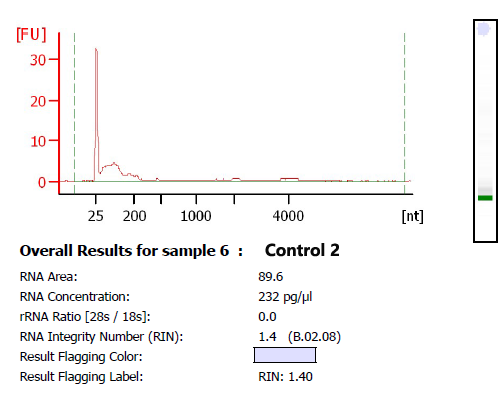


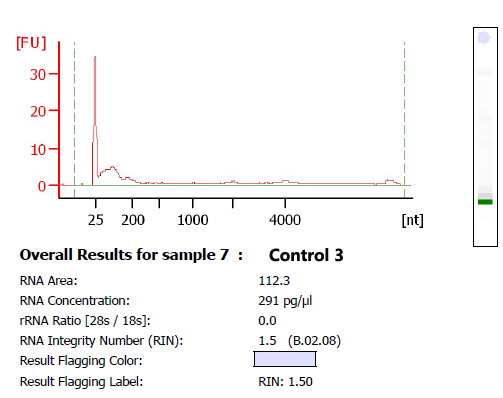


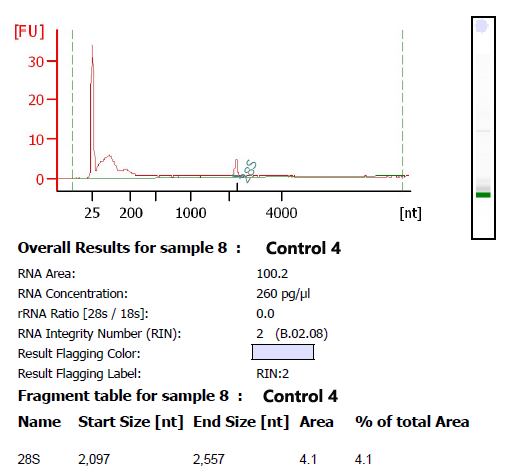

Supplement: Supplementary file 2 — Additional file 2: Supplement Figure 1. [file 40246_2021_354_MOESM2_ESM.docx]
